# Correction: Low-cost solution for rodent home-cage behaviour monitoring

**DOI:** 10.1371/journal.pone.0221884

**Published:** 2019-08-26

**Authors:** The PLOS ONE Staff

## Notice of republication

Incorrect versions of Figs 1, 6, and 7 were published in error. The publisher apologizes for the errors. This article was republished on August 15, 2019 to correct for these errors. Please download this article again to view the correct version. The originally published, uncorrected article and the republished, corrected articles are provided here for reference.

## Supporting information

S1 File Originally published, uncorrected article. (PDF)

S2 File Republished, corrected article. (PDF)
